# *Phyllanthus rufuschaneyi*: a new nickel hyperaccumulator from Sabah (Borneo Island) with potential for tropical agromining

**DOI:** 10.1186/s40529-018-0225-y

**Published:** 2018-03-27

**Authors:** Roderick Bouman, Peter van Welzen, Sukaibin Sumail, Guillaume Echevarria, Peter D. Erskine, Antony van der Ent

**Affiliations:** 1 Naturalis Biodiversity Center, Botany, 2300 RA The Netherlands; 2 Hortus Botanicus, Leiden University, Leiden, 2311 GJ The Netherlands; 3 Institute of Biology Leiden, Leiden University, Leiden, 2300 RA The Netherlands; 4 Herbarium, Sabah Parks, Kota Kinabalu, Sabah Malaysia; 5 Laboratoire Sols et Environnement, Université de Lorraine, INRA, Nancy, 54000 France; 6 Centre for Mined Land Rehabilitation, Sustainable Minerals Institute, The University of Queensland, St Lucia, QLD 4072 Australia

**Keywords:** *Epicephala* pollination, Nickel hyperaccumulation, Phyllanthaceae, *Phyllanthus* subgenus *Gomphidium*, Sabah

## Abstract

**Background:**

Nickel hyperaccumulator plants are of much interest for their evolution and unique ecophysiology, and also for potential applications in agromining—a novel technology that uses plants to extract valuable metals from soil. The majority of nickel hyperaccumulators are known from ultramafic soils in tropical regions (Cuba, New Caledonia and Southeast Asia), and one genus, *Phyllanthus* (Phyllanthaceae), is globally the most represented taxonomic entity. A number of tropical *Phyllanthus*-species have the potential to be used as ‘metal crops’ in agromining operations mainly because of their ease in cultivation and their ability to attain high nickel concentrations and biomass yields.

**Results:**

One of the most promising species globally for agromining, is the here newly described species *Phyllanthus rufuschaneyi*. This species can be classified in subgenus *Gomphidium* on account of its staminate nectar disc and pistillate entire style and represents the most western species of this diverse group. The flower structure indicates that this species is probably pollinated by *Epicephala* moths.

**Conclusions:**

*Phyllanthus rufuschaneyi* is an extremely rare taxon in the wild, restricted to Lompoyou Hill near Kinabalu Park in Sabah, Malaysia. Its utilization in agromining will be a mechanism for conservation of the taxon, and highlights the importance of habitat and germplasm preservation if rare species are to be used in novel green technologies.

## Background

Whereas the great majority of plants growing on naturally nickel (Ni) rich ultramafic soils exclude it from uptake and translocation, a minority of plants display a highly unusual response with enhanced uptake and transfer to the shoots (Reeves 2003; Van der Ent et al. 2013a). These plants are called ‘hyperaccumulators’ and they have the ability to accumulate trace elements to extreme concentrations in their living tissues (Jaffré et al. 1976; Van der Ent et al. 2013a). The Ni concentrations in some species can reach up to 16.9 Wt% in the phloem sap (Van der Ent and Mulligan 2015). Although there are over 400 known Ni hyperaccumulators species (> 0.1 Wt% shoot dry weight), there are just ca. 50 *hypernickelophores* (e.g. hyperaccumulator species with > 1 Wt% Ni shoot dry weight) known globally (Reeves 2003; Reeves et al. 2017). Hyperaccumulator plants can be used as ‘metal crops’ in agromining (phytomining) operations to generate metal-rich biomass for commercial gain (Chaney et al. 1998; Van der Ent et al. 2015a). This innovative approach enables access to resources not accessible by conventional mining techniques such as abundant low-grade sources of valuable elements (Li et al. 2003; Van der Ent et al. 2015a). Agromining can also benefit local communities, by providing new income opportunities for farmers in developing countries (Bani et al. 2015; Chaney et al. 2018). The greatest potential for agromining is in tropical regions (Cuba, New Caledonia and Southeast Asia) where some of the world’s largest low-grade nickel sources are located (Van der Ent et al. 2013b).

On a global scale, Ni hyperaccumulation occurs most frequently in the order Malpighiales, particularly in the families Dichapetalaceae, Phyllanthaceae, Salicaceae and Violaceae.

The Phyllanthaceae has the greatest numbers of hyperaccumulators with representatives in the genera *Actephila* Blume, *Antidesma* L., *Breynia* J.R.Forst. & G.Forst., *Cleistanthus* Hook.f. ex Planch., *Glochidion* J.R.Forst. & G.Forst. and *Phyllanthus* L. The latter is pantropical and the most speciose genus of the family with over 800 species globally (Govaerts et al. 2000; Kathriarachchi et al. 2006; Bouman et al. under review). Due to its great diversity in morphology, *Phyllanthus* is currently classified in many subgenera and (sub)sections, which were often former separate genera. The genus is characterized by its unisexual flowers, the absence of petals and a characteristic branching system called phyllanthoid branching (see Fig. 2a; Webster 1956). Species with this particular type of branching have deciduous, plagiotropic branchlets that are subtended by reduced scale-like leaves (cataphylls) (Webster 1956). Normal leaves and flowers are only found on the plagiotropic branchlets. This branching system has been lost several times (Kathriarachchi et al. 2006) and in species with non-phyllanthoid branching, leaves can be found on all axes and the branchlets are not deciduous and subtended by normal leaves. The genus *Phyllanthus* is currently paraphyletic with the genera *Breynia*, *Glochidion and Synostemon* F.Muell. nested within (Kathriarachchi et al. 2006; Pruesapan et al. 2012). Discussions on how to resolve the paraphyly of the genus *Phyllanthus* are still ongoing (see Hoffmann et al. 2006; Van Welzen et al. 2014). One of the most speciose subgenera is subgenus *Gomphidium* (Baill.) G.L.Webster. Species in the subgenus are characterised by phyllanthoid branching in combination with flowers with biseriate sepals, stamens with free filaments (but connate in one section) and three duplex nectar glands (sometimes divided and then 6 or absent). The phylogenetic position of subgenus *Gomphidium* was not consistent between the markers used in the study of Kathriarachchi et al. (2006) and requires further study.

Major centres of diversity for *Phyllanthus* are in New Caledonia with over 100 species of which 15 species are Ni hyperaccumulators (Kersten et al. 1979; Schmid 1991; Jaffré et al. 2013), in Cuba with at least 40 species of which 17 species are Ni hyperaccumulators (Leon and Alain 1953; Reeves et al 1996), and in the Malesian Region about 100 species of which 5 species are Ni hyperaccumulators (Van der Ent et al. 2015a, b, c; Wu et al. 2016; Galey et al. 2017).

A number of taxa in the genus *Phyllanthus* are among the most promising ‘metal crops’ due to their fast growth and other favourable growth characteristics, including easy propagation and pest resistance (Nkrumah et al. 2016). Some *Phyllanthus*-species also reach some of the highest Ni concentrations known in any hyperaccumulator plants, with 3.8 Wt% in the leaves of *P. serpentinus* S.Moore from New Caledonia (Jaffré 1977; Kersten et al. 1979; as *P. favieri* M.Schmid), 3.9 Wt% in the leaves of *Phyllanthus insulae*-*japen* Airy Shaw from Indonesia (Reeves 2003), and 6 Wt% in the leaves *P.* × *pallidus* C.Wright ex Griseb. from Cuba (Reeves et al. 1996).

Further study of other genera within the Phyllanthaceae continues to yield new Ni hyperaccumulator records, such as in the genus *Antidesma* (Nkrumah et al. 2018), and even new species that are hyperaccumulators, such as the recently described *Actephila alanbakeri* Welzen & Ent (Van der Ent et al. 2016a). Kinabalu Park is the world’s most species-rich hotspot with over 5000 species in 1000 genera and 200 families recorded to date (Beaman and Beaman 1990; Beaman 2005) of which 2542 plant species have been found on the ultramafic soils inside the Park (Van der Ent et al. 2015c). In Sabah, a total of 8 species of *Phyllanthus* occur, of which two are known Ni hyperaccumulators: *P. balgooyi* Petra Hoffm. & A.J.M.Baker which can accumulate up to 1.6 Wt% Ni in the leaves and up to 16.9 Wt% Ni in the phloem sap and the here newly described *Phyllanthus rufuschaneyi* (Hoffmann et al. 2008; Van der Ent and Mulligan 2015; Mesjasz-Przybylowicz et al. 2016). *Phyllanthus balgooyi*, also occurs in the Philippines, in addition to *Phyllanthus securinegioides* Merr. (which can accumulate up to 3.5 Wt% in the leaves), and a third Ni hyperaccumulator species from the genus, *P. erythrotrichus* C.B.Rob. which can accumulate up to 1.1 Wt% Ni in the leaves (Baker et al. 1992; Quimado et al. 2015). The extreme levels of Ni accumulation in these species poses important questions about the ways in which these plants take up, transport and store Ni, while avoiding the potential effects of metabolic toxicity. In leaves, Ni appears to be associated mainly with organic acids, such as citrate, malate and malonate (Kersten et al. 1980; Homer 1991; Montargès-Pelletier et al. 2008; Van der Ent et al. 2017).

**Fig. 1:**
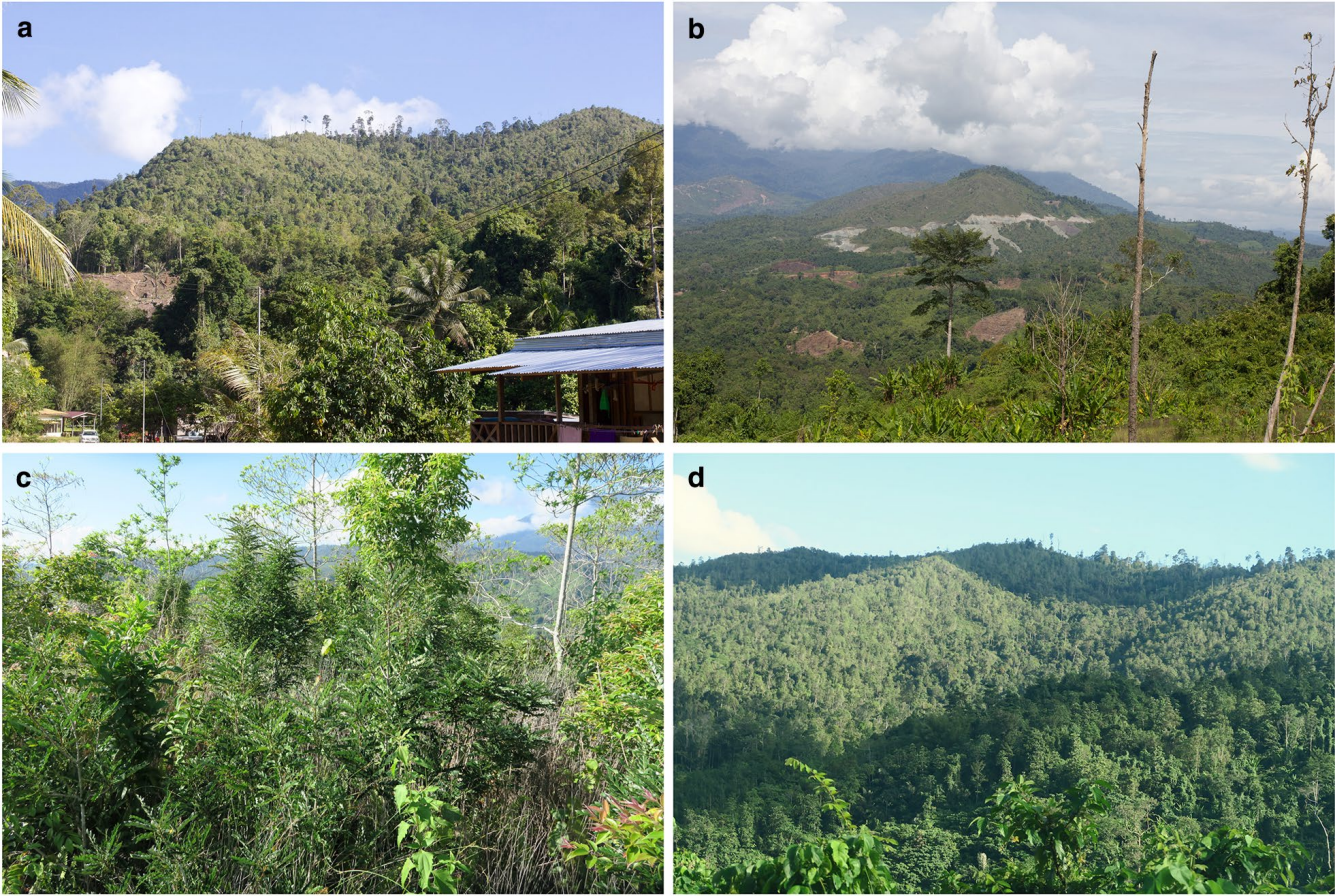
Different aspects of the habitat of *P. rufuschaneyi* in Sabah, Malaysia. **a** Lompoyou Hill seen from Nalumad village; **b** Garas—the eastern end of Lompoyou Hill, the outcropping ultramafic (serpentinite) bedrock is clearly visible in the road cuts; **c** *Phyllanthus rufuschaneyi* growing in situ on Lompoyou Hill; **d** the summit of Lompoyou Hill with secondary scrub and dead standing trees after forest fires. Images by A. van der Ent

In early 2013, a hitherto unknown species of *Phyllanthus* was planted in the local garden by staff at the Monggis substation of Kinabalu Park. Spot-testing with dimethyl-glyoxime-impregnated paper revealed it to be a strong Ni hyperaccumulator, which was subsequently confirmed through Inductively Coupled Plasma Atomic Emission Spectroscopy (ICP-AES) on acid-digested samples of leaves in the laboratory (first reported in Van der Ent et al. 2013c). The plant was collected from an unknown location near the Kinabalu Park boundary and could not be re-collected at the time. In 2015, the taxon was ‘re-discovered’ during fieldwork on Lompoyou Hill, approximately 12 km from Monggis substation, where it was locally abundant (Fig. 1). The taxon is of significant scientific interest because of the extremely high levels of Ni accumulation, reaching up to 2.8 Wt% Ni in leaves and 1.8 Wt% in the phloem tissue (Van der Ent and Mulligan 2015). Following its discovery, the ecophysiology was studied in detail, which revealed that Ni is mainly concentrated in the phloem in roots and stems, and in the leaves in the epidermis (Van der Ent et al. 2017). Apart from scientific interest, the taxon has great potential for agromining, which was studied in a pioneering nursery pot experiment and field trial (Nkrumah et al. 2016). This experimental work demonstrated that *P. rufuschaneyi* has advantageous characteristics for utilization as a ‘metal crop’ including fast growth rate, easy re-growth after coppicing, tolerance for exposed conditions on eroded soils and high Ni accumulation in its whole biomass. Taken together, *P. rufuschaneyi* has the best combination of characteristics of any tropical ‘metal crop’ presently known. The bio-ore (e.g. ashed biomass) of *P. rufuschaneyi* contains up to 12.7 Wt% Ni and the extractive hydrometallurgy of this material was studied for producing high-purity Ni salts for the electrochemical industry (Vaughan et al. 2017). At first, this plant was only known as *Phyllanthus* cf. *securinegioides* Merr. (and reported as such in Van der Ent et al. 2015b, 2016b, 2017), because of its superficial resemblance to this species from the Philippines. However, after examination of the literature and other specimens, we conclude that it is a new species that is of great interest for its taxonomic position and its qualities as a hyperaccumulator. Here we describe this taxon and provide information about its taxonomic relations, distribution, ecology, hyperaccumulation properties, and conservation status.

## Methods

### Taxonomical investigation

The material studied comprised of herbarium specimens loaned from the Sabah Parks Herbarium (SNP) in Sabah, Malaysia. Descriptions were made using standard taxonomical techniques and morphological terminology follows Beentje (2016).

The IUCN conservation status was assessed applying the IUCN Red List Categories (IUCN 2001) that considers (i) extent of occurrence (EOO), and (ii) area of occurrence (AOO) in order to generate applicable IUCN threat categories.

### Collection of plant samples for chemical analysis

Plant tissue samples (leaves, wood, bark, flowers) for bulk chemical analysis were collected in the habitat (6°06′29.6″N 116°47′36.7″E) near Kinabalu Park in Sabah, Malaysia. These samples were dried at 70 °C for 5 days in a drying oven and subsequently packed for transport to Australia and gamma irradiated at Steritech Pty. Ltd. in Brisbane following Quarantine Regulations in Australia. The dried plant tissue samples were subsequently ground and digested using 4 mL HNO_3_ (70%) and 1 mL H_2_O_2_ (30%) in a microwave oven (Milestone Start D) for a 45-min programme and diluted to 30 mL with ultrapure water (Millipore 18.2 MΩ cm at 25 °C) before analysis with ICP-AES (Varian Vista Pro II) (Huang et al. 2004). The elemental concentrations originate from previously reported data (Van der Ent and Mulligan 2015; Van der Ent et al. 2015b, 2017) augmented with new data from plant tissue samples collected for this study.

## Results

### Taxonomic treatment

**Fig. 2:**
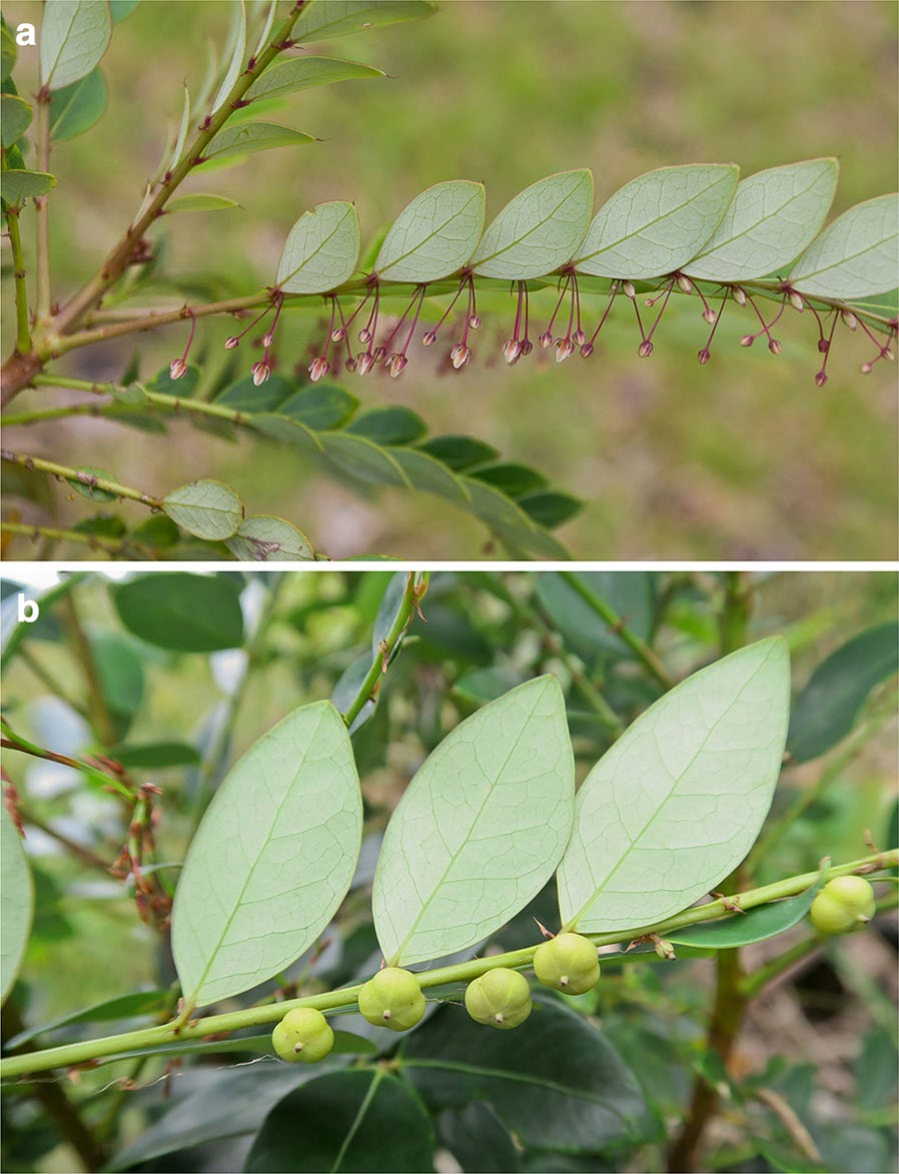
Detail of *P. rufuschaneyi* plants. **a** Inflorescences of *P. rufuschaneyi*, note the difference between main stem and side stem with at the base small structures that signal phyllanthoid branching; **b** fruit capsules of *P. rufuschaneyi*. Images by A. van der Ent

**Fig. 3:**
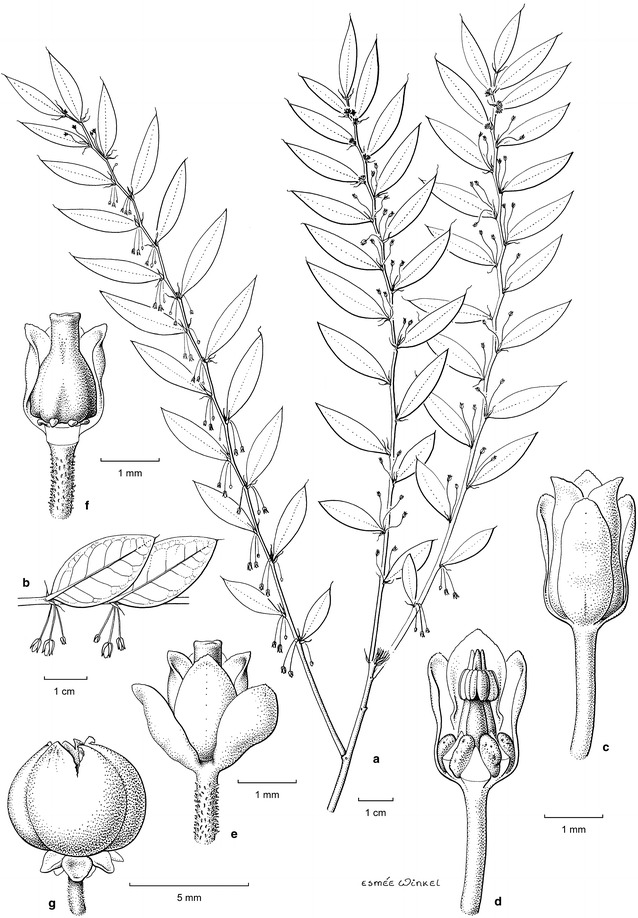
*Phyllanthus rufuschaneyi* Welzen, R.W.Bouman & Ent: **a** a branch with only scars of cataphylls and cataphyllary stipules present at the base of branchlets as these are caducous (drawn from herbarium specimen with leaves glued sideways and staminate flowers sometimes upright instead of hanging); **b** detail of sidebranch with leaves and staminate flowers in natural position; **c** staminate flower; **d** staminate flower with part of sepals removed showing disc glands and androecium; **e** pistillate flower; **f** pistillate flower with part of sepals removed showing disc glands and ovary; **g** fruit (**a**, **c**, **d** *Daim Endau 225*; **b** *Lomudin Tadon g257*; **e**, **f** *SNP 32987*; **g** *Lomudin Tadon 257*; all SNP). Drawing by Esmée Winkel (2017)

**Fig. 4:**
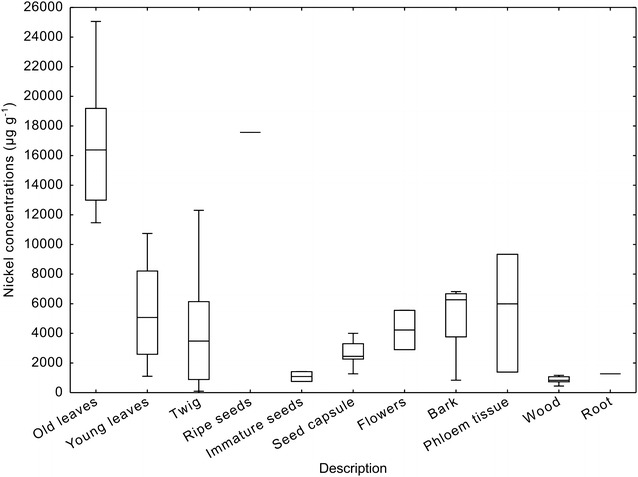
Elemental concentration in various plant parts of *P. rufuschaneyi*. Data from Tables 1 and 2

*Phyllanthus rufuschaneyi* Welzen, R.W.Bouman and Ent, *sp. nov.*—TYPE: MALAYSIA. Sabah, near Kampong Nalumad, eastern boundary Kinabalu Park, Lompoyou Hill, Antony Van der Ent et al. SNP 32987! (holo SNP; iso L). Paratype: SNP 22039!, Lompoyou Hill, Sabah, Malaysia (Figs. 2, 3, 4).

This species is most similar to *P. securinegoides* from the Philippines, from which it can be distinguished by its smaller leaves, staminate flowers with connate filaments and pistillate flowers with connate tubular stigmas.

Shrub to tree(let), up to 6 m high, monoecious, phyllanthoid branching present, main branches often hollow. *Indument* generally absent, on some parts asperities (stiff, short, papillae-like hairs) on orthotropic and plagiotropic branches and sometimes petioles and pedicels. *Plagiotropic branches* 1‒1.5 mm wide, not flattened, with 2 narrow longitudinal wings, to 0.3 mm wide, asperities usually present between wings. *Stipules* ovate, 1.5‒6 by 0.8‒1 mm, persistent, becoming brown when dry, basally eared, ears sometimes elongated. *Leaves* distichous, simple, dimorphous (reduced on orthotropic branch to cataphylls (scale leaves), not reduced on plagiotropic branches). *Cataphylls* on main trunk below branches, stipule-like, ovate to triangular, c. 3 mm long, early caducous. *Leaves on plagiotropic branches*: petiole more or less dorsoventrally flattened, 1‒1.5 mm long, glabrous or with asperities, transversely wrinkled when dry; blade ovate to elliptic, 1.6‒3.7 (without mucro) by 0.5‒1.2 cm, 2.2‒3.7 times longer than wide, coriaceous, base attenuate, asymmetric, margin entire, somewhat thickened, slightly revolute, apex rounded to acuminate, mucronate up to 5.2 mm, mucron usually breaking off, upper and lower surface glabrous, smooth, lower surface glaucous; venation pinnate, above hardly visible, slightly raised underneath, secondary veins 6‒8 per side, looped and closed near margin, higher order veins reticulate. *Inflorescences* consisting of unisexual or bisexual fascicles, flowers 1‒4 per node; staminate flowers single or a few, generally in lower axils but sometimes at end of branches, usually together with a single pistillate flower; latter also single in upper axils. *Staminate flowers* 1.4‒2 mm diameter, remaining closed, actinomorphic; pedicel slender, 6.7‒12 mm long, apically somewhat thickened; sepals 6, upright, tightly packed, margin entire, apex rounded; outer three smaller (when young) to slightly longer than inner ones, 1.9‒2 by 0.9‒1 mm, central part thickened, pink, margins and upper half thin, white to whitish pink; inner 3 1.4‒2.3 by 1.3‒1.4 mm, lower part almost nail-like, central midrib area thickened and darker coloured, basal central part inside attached to androphore via a ^-like structure; disc lobes 6, paired, vertical, kidney-like, c. 0.6 mm long, greyish blue when dry, attached to broad part of flower receptacle, smooth; stamens 3, filaments connate, c. 1 mm high, broadly cone-shaped, apically with erect anthers, these elliptic, c. 0.7 by 0.4 mm, opening extrorse via lengthwise slits, apically on connective a c. 0.3 mm long slender appendix. *Pistillate flowers* 1.4‒1.6 mm diameter, actinomorphic; pedicel 1‒2 mm long, round, glabrous or with asperities; sepals 6, tight to ovary in flower, spreading in fruit, base very thickened, attached to receptacle, margin entire, outer 3 slightly smaller than inner 3, elliptic, 1.7‒2 by c. 1 mm, apex truncate to erose, inner 3 elliptic, c. 2 by 1.2‒1.6 mm, apex rounded; disc lobes 6, small, globose, less than 0.2 mm in diameter; gynophore c. 0.3 mm high, ovary ovoid, c. 1.3 by 1.1‒1.3 mm, 3-locular, 2 ovules per locule, smooth, glabrous, three stigmas united into a cone of 0.8‒1 mm high, apically somewhat erose (slightly split stigmas) and hollow inside. *Fruits* c. 7.5 mm in diameter, c. 4.5 mm high, opening completely septicidal and partly to completely loculicidal, exocarp separating from meso- and endocarp, wall thin, woody when dry, smooth, glabrous; columella broadly triangular, 2‒2.3 mm high. *Seed* ovoid-triangular, c. 2.8 by 1.8 mm, brown, smooth, not seen mature.

### Etymology

The specific epithet “*rufuschaneyi*” honours Dr. Rufus L. Chaney (b. 1942), an agronomist who is widely credited for inventing phytomining (agromining) (Chaney 1983), leading to the technology being patented (Chaney et al. 1998). Dr. Chaney has worked for 47 years at the USDA Agricultural Research Service (USA) on risk assessment for metals in soils and crops, and the food-chain transfer and bioavailability of soil and crop metals to humans. He published over 490 publications and won the Gordon Award for Lifetime Achievement and Excellence in Phytoremediation Research. The fact that *P. rufuschaneyi* is the most promising tropical Ni ‘metal crop’ presently known, makes this recognition fitting.

### Phenology and pollination

The species flowers and fruits all year round. Especially young plants growing in open habitats flower profusely on many branches. Flowering is less frequent when plants grow under more shaded conditions in developing forest. Many *Phyllanthus* species are pollinated by moths of the genus *Epicephala* Meyrick. Females of these moths actively pollinate the flowers and then deposit eggs into the floral ovaries, after which the larvae consume some of the developing seeds (Kawakita 2010). *Phyllanthus rufuschaneyi*, like all other species in subgenus *Gomphidium*, is likely pollinated by *Epicephala* moths (Kato and Kawakita 2017) as the staminate flowers are highly closed (inner sepals even grown together with the broad androphore) and possess vertical anthers, the pistillate flowers have grown together, cone-like, non-opening stigmas. Although pollination has not been specifically studied in this species, *Epicephala* larvae were observed in the fruit capsules of *P. rufuschaneyi* in the field. The similarity with *Glochidion* flowers, also pollinated by *Epicephala* moths, is remarkable. The co-evolution and high specificity for *Epicephala*-species for *Phyllanthus*-species has been linked to the extensive diversification of this genus in New Caledonia (Kawakita and Kato 2004).

### Distribution, habitat and ecology of *Phyllanthus rufuschaneyi*

*Phyllanthus rufuschaneyi* is known only from two populations; one (very small) population at the foot of Bukit Hampuan, and another larger population on Lompoyou Hill approximately 5 km from the first population. The habitat in both localities is open secondary scrub that has been affected by recurring forest fires (Fig. 1). Lompoyou Hill is close to the villages of Nalumad and Pahu. The hill (400 m asl) has been burnt at least once as a result of an uncontrolled forest fire in 1998. Prior to burning, the site was already disturbed by logging. The area has a short and open scrub community (dominated by shrubs 1–3 m tall) with pioneer species such as *Macaranga kinabaluensis* Airy Shaw (Euphorbiaceae). In this habitat type several other Ni hyperaccumulator plant species occur, including *Phyllanthus balgooyi*, *Actephila alanbakeri*, *Mischocarpus sundaicus* Blume (Sapindaceae), and *Xylosma luzonensis* Clos (Salicaceae). The local conditions are xeric, and the soils are shallow and heavily eroded with limited amounts of organic matter. In pot experiments *P. rufuschaneyi* responded negatively to increasing organic matter amendments (Nkrumah et al. 2017). *Phyllanthus rufuschaneyi* occurs exclusively on these young eroded soils (hypermagnesian Cambisols) that occur at low elevation (700 m asl) on strongly serpentinised bedrock. These soils have extremely high magnesium (Mg) to calcium (Ca), circum-neutral pH, and high available Ni as a result of the disintegration of phyllosilicates and re-sorption onto secondary iron (Fe)-oxides or high-charge clays (Echevarria 2018). In Sabah, Ni hyperaccumulator plant species are restricted to these soils with a pH > 6.3 and relatively high total soil Ni concentrations > 630 μg g^−1^ (Van der Ent et al. 2016b).

### Elemental concentrations in the plant tissues

Bulk elemental concentrations of macro-elements (mainly essential nutrients) are given in Table 1; Fig. 4. Aluminium (Al) concentrations are uniformly low in all plant parts, but highest in the flowers (up to 370 μg g^−1^). Calcium is high to extremely high in many parts of the plant, especially in the old leaves (up to 1.09 Wt%), in the bark (up to 2.34 Wt%) and in the twigs (up to 2.59 Wt%). Potassium (K) concentrations are remarkably high for a plant species growing on severely K-deficient soils. The highest K concentrations are in the old leaves (up to 1.34 Wt%) and twigs (up to 1.37 Wt%), and the lowest in the roots (53 μg g^−1^). Magnesium concentrations are particularly high in the immature seeds (mean of 4931 μg g^−1^). Sodium (Na) concentrations are unremarkable, but highest in the young leaves with up to 2325 μg g^−1^ (mean is 390 μg g^−1^). Finally, sulfur (S) concentrations vary widely with the lowest concentrations in the roots (81 μg g^−1^) and the highest in the immature seeds (up to 3290 μg g^−1^).

**Table 1:** Elemental concentration (macro elements: Al, Ca, K, Mg, Na, P, S) ranges and means in parentheses

Plant part	*N* samples	Al	Ca	K	Mg	Na	P	S
Flowers	2	20–370 [195]	1020–3330 [2180]	431–4600 [2520]	2810–3630 [3220]	65–301 [183]	392–1070 [732]	347–904 [625]
Fruit capsule	5	9.0–70 [27]	672–4160 [2410]	2700–5920 [4600]	563–1800 [1080]	38–305 [123]	191–1240 [738]	757–1390 [972]
Immature seeds	2	26–296 [161]	4340–4480 [4410]	6010–6090 [6050]	2740–7120 [4930]	70–661 [365]	3330–3880 [3610]	1940–3290 [2610]
Ripe seeds	1	85	9460	4570	4100	603	1856	2380
Young leaves	12	1.0–52 [20]	124–12,200 [4100]	89–19,500 [7250]	352–6900 [3020]	1.9–2320 [390]	34–5180 [1010]	152–3610 [1440]
Old leaves	21	12–69 [30]	2416–10900 [4730]	3101–13,400 [5760]	1060–5500 [3140]	30–525 [120]	430–3220 [789]	1140–3100 [1660]
Bark	7	14–71 [43]	92–23,400 [10,870]	45–6240 [3750]	361–2160 [728]	19–530 [213]	16–505 [332]	108–1127 [749]
Phloem tissue	3	29–95 [51]	129–36,400 [16,500]	84–8420 [4970]	13–1360 [975]	26–384 [222]	30–339 [180]	152–1100 [735]
Twigs	14	1.0–71 [21]	130–25,900 [5200]	246–13,700 [5520]	109–5280 [966]	8.8–856 [313]	16–1730 [539]	53–1720 [610]
Wood	7	2.8–23 [14]	125–1296 [762]	78–2725 [1585]	85–249 [150]	5.0–483 [200]	23–791 [359]	48–506 [306]
Roots	1	91	125	53	382	31	40	81

All values provided in μg g^−1^

**Fig. 5:**
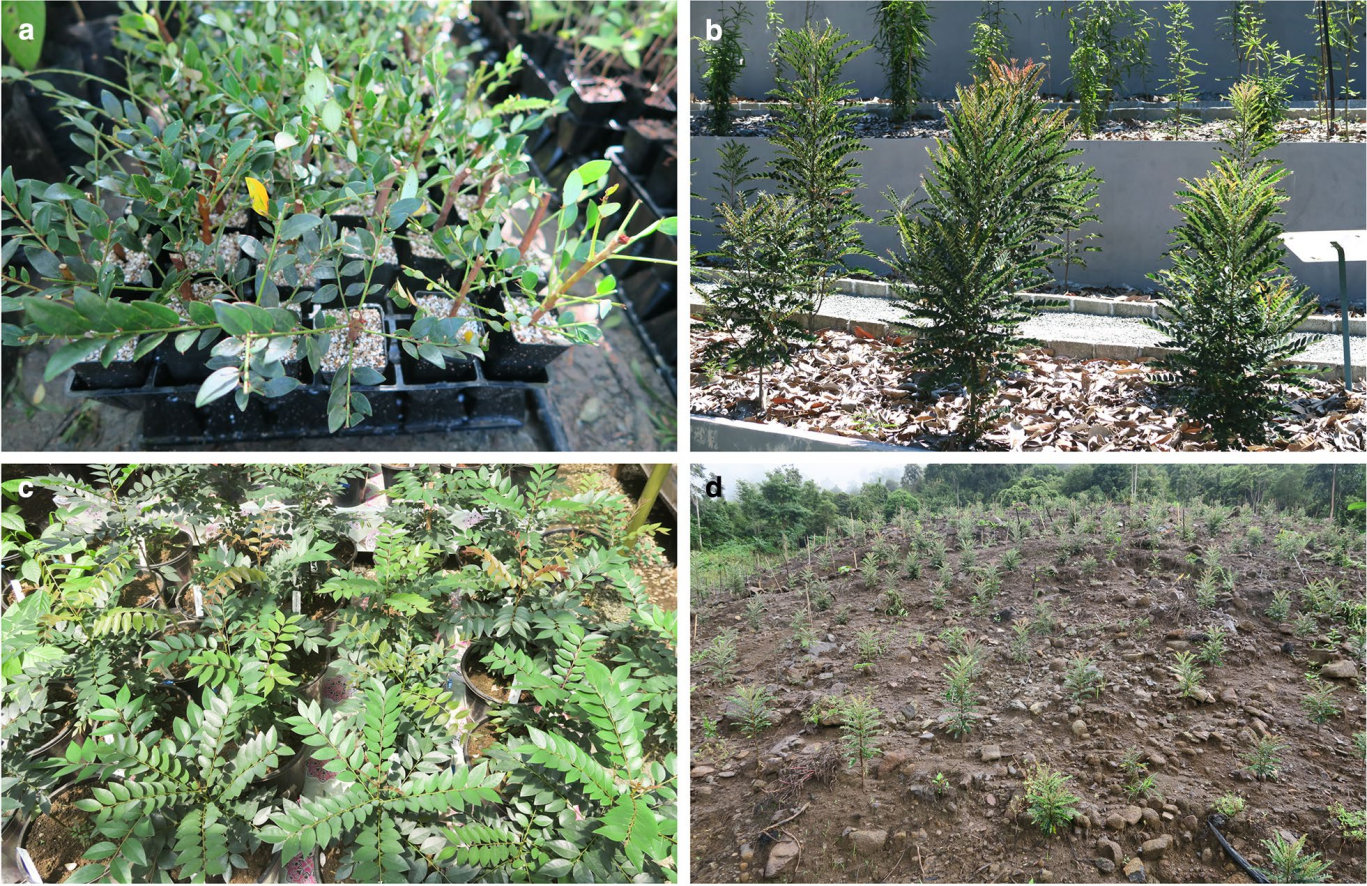
Agromining growth trials using *P. rufuschaneyi* in Sabah, Malaysia. **a** Mass propagation of *P. rufuschaneyi* using cutting grown in perlite; **b** *Phyllanthus rufuschaneyi* shrubs planted out in the ‘Hyperaccumulator Botanic Garden’ of Sabah Parks; **c** plant nutrition growth experiment using *P. rufuschaneyi* at Monggis substation; **d** full-scale (1.5 ha) agromining field trial using *P. rufuschaneyi* near Pahu village 6 months after planting

Bulk elemental concentrations of trace-elements (transition group elements) are given in Table 2. Cobalt (Co) concentrations are the highest in the young leaves with up to 198 μg g^−1^, but are comparatively low in relation to Ni concentrations. The mean Co:Ni quotient is 1:532, evidencing highly selective uptake of Ni over Co, compared to soil concentrations of these elements (generally 10:1 Ni to Co). Chromium (Cr) concentrations are universally low, and this element is clearly excluded from uptake. Copper (Cu) concentrations have a narrow range between 0.7–17 μg g^−1^, with mean values of 6.7 and 4.7 μg g^−1^ in young and old leaves respectively. Iron concentrations are more variable, but generally low (maximum of 564 μg g^−1^ in the phloem tissue). Manganese (Mn) concentrations too are low in all plant parts, with the highest concentrations on the old leaves (up to 461 μg g^−1^). Nickel concentrations are the highest in the old leaves with up to 2.50 Wt% (mean of 1.65 Wt%) and somewhat lower in the young leaves (up to 1.08 Wt% and a mean of 5390 μg g^−1^). Nickel concentrations are also high in the twigs (up to 1.23 Wt%) and in the phloem tissue (up to 0.93 Wt%). It is remarkable that the reproductive organs (flowers, fruit capsule and seeds) are highly Ni-enriched. The seeds contain 1.76 Wt% Ni and the flowers on average 0.43 Wt% Ni. The elemental fractionation of Ni in the different plant parts is depicted in Fig. 5. Finally, zinc (Zn) concentrations are unremarkable with on average 85 μg g^−1^ in the old leaves.

**Table 2:** Elemental concentration (trace elements: Co, Cr, Cu, Fe, Mn, Ni, Zn) ranges and means in parentheses

Plant part	*N* samples	Co	Cr	Cu	Fe	Mn	Ni	Zn
Flowers	2	16–104 [60]	14–17 [16]	6.5–9.5 [8.0]	15–24 [20]	34–945 [490]	2905–5560 [4230]	20–100 [60]
Fruit capsule	5	4.3–31 [13]	2.3–17 [8.4]	2.2–10 [5.9]	8.0–30 [20]	11–83 [35]	1274–4001 [2660]	9.0–22 [17]
Immature seeds	2	23–89 [56]	15–28 [22]	8.5–13 [11]	30–43 [36]	58–64 [61]	754–1421 [1090]	26–34 [30]
Ripe seeds	1	3	114	14	131	187	17,570	182
Young leaves	12	3.0–198 [46]	1.3–66 [16]	0.1–17 [6.7]	9–241 [63]	21–182 [93]	1105–10,750 [5388]	10–244 [52]
Old leaves	21	12–75 [31]	1.2–13 [6.9]	0.9–11 [4.7]	22–90 [46]	95–461 [170]	11,470–25,060 [16,490]	45–249 [85]
Bark	7	4.4–28 [14]	2.3–13 [6.3]	1.0–10 [4.5]	22–429 [85]	33–89 [66]	842–6820 [5260]	18–83 [69]
Phloem tissue	3	12–49 [27]	6.3–15 [10]	1.3–6.6 [4.1]	37–564 [220]	62–126 [103]	1390–9340 [5570]	28–191 [100]
Twigs	14	1.9–36 [16]	2.4–42 [8.0]	0.5–33 [6.2]	4.0–40 [17]	5.0–259 [72]	99–12,300 [4330]	5.1–103 [49]
Wood	7	0.8–15 [5.0]	1.9–4.7 [3.4]	0.7–6.3 [2.5]	3.7–10 [5.7]	5.9–18 [11]	443–1170 [862]	4.8–14 [10]
Roots	1	26	6.5	0.7	512	164	1272	24

All values provided in μg g^−1^

### Conservation status

The habitat of *P. rufuschaneyi* is outside any protected areas, in patches of remaining scrub in an area devastated by recurring forest fires. The restriction of this species to just one main population (the second population is very small) and the small area of occupancy (< 10 km^2^) means that this species is sensitive to disturbances which could ultimately lead to its extinction. Therefore, the species can be classified as Critically Endangered (CR) on the basis of IUCN Red List Criteria (Version 3.1: IUCN 2001) considering Criterion B. Geographic range; B2. Area of occupancy estimated to be less than 10 km^2^, and a. Severely fragmented or known to exist at only a single location; b. Continuing decline in (i) extent of occurrence; and (iii) area, extent and/or quality of habitat.

## Discussion

The taxonomic position of *P. rufuschaneyi* is noteworthy as it represents the most western species of its subgenus. It is placed in *Phyllanthus* subgenus *Gomphidium* section *Nymania* (K.Schum) J.J.Sm. on account of its three paired staminate disc glands and its connate stamens (Fig. 3c). Section *Nymania* is mainly distributed in Papua New Guinea, but a few species also occur in the Philippines. *Phyllanthus rufuschaneyi* is most similar to *P. securinegioides*, however, it differs in its significantly smaller leaves, its smaller fascicled inflorescences with pinkish flowers (Fig. 1a; vs larger fascicles with yellow flowers in *P. securinegioides*), the completely fused anther filaments (Fig. 3c) and the fused style (Fig. 3e). The other Philippine species of *Gomphidium*, *P. apiculatus* Merr., *P. ramosii* Quisumb., *P. glochidioides* Elmer and *P. cordatulus* Rob., all have staminate flowers with free stamens. Though *P. cordatulus* has a similarly fused style of c. 1.3 mm long, it is spreading for about the same distance, whereas the style of *P. rufuschaneyi* is only fused for up to 1 mm and slightly erose at the end. The style is quite prominent and emerges out of the pistillate flowers (Fig. 3d, e). The other species of *Phyllanthus* occurring in Borneo are from other subgenera and differ either in their branching system (e.g. phyllanthoid vs non-phyllanthoid in subgenus *Macraea* (Wight) Jean F.Brunel) or in the morphology of their flowers (e.g. no difference between inner and outer sepals in subgenera *Emblica* (Gaertn.) Kurz, *Kirganelia* (A.Juss.) Kurz and *Eriococcus* (Hassk.) Croizat & Metcalf). The structure on top of the anther connective is slightly reminiscent of the apiculate anthers in subgenus *Phyllanthodendron* (Hemsl.) G.L.Webster. However, *P. rufuschaneyi* does not have the characteristic ligulate nectar disc and apiculate anthers that are also found in subgenus *Gomphidium*. Nickel hyperaccumulators are found in several subgenera within the genus *Phyllanthus*, which suggests that it evolved several times.

The restricted distribution of *P. rufuschaneyi* cannot be easily explained. In Sabah, Ni hyperaccumulators are restricted to circum-neutral soils with relatively high phytoavailable calcium, magnesium and Ni concentrations with at least 20 μg g^−1^ carboxylic acid extractable Ni or 630 μg g^−1^ total nickel, and a soil pH > 6.3 (Van der Ent et al. 2016b). However, these types of Cambisols derived from serpentinized bedrock are relatively widespread in Sabah, but *P. rufuschaneyi* has not been found elsewhere despite extensive fieldwork on all major ultramafic outcrops in Sabah (Van der Ent et al. 2015b). Although some Ni hyperaccumulators are comparatively common (*P. balgooyi*, *Psychotria sarmentosa* Blume, Rubiaceae, *Xylosma luzonensis*) on several ultramafic outcrops in Sabah, the situation of *P. rufuschaneyi* mirrors that of *Actephila alanbakeri*, which is also restricted to a single site at the same location (Van der Ent et al. 2016a). Experience in cultivation shows that *P. rufuschaneyi* is highly shade-intolerant and requires exposed conditions with minimal light competition (Fig. 5). It suffers from fungal infections when grown under shaded and moist conditions, and stops flowering. The dependence on a specific pollinator that is characteristic for the *Phyllanthus*-*Epicephala* mutualism and limited dispersal capabilities might be possible explanations why this species is not more widespread in Borneo.

*Phyllanthus rufuschaneyi* has particularly attractive characteristics for cultivation as ‘metal crop’ in agromining operations. These favourable properties include its multi-stemmed habit, the rapid re-growth after coppicing, and high Ni concentrations in woody parts of the biomass. Other *Phyllanthus*-species, such as *P. balgooyi*, albeit having equally high foliar Ni concentrations, have lower growth rates and do not tolerate open and exposed conditions on bare soils. A major uncertainty currently pertains to the effective mass-propagation of *P. rufuschaneyi,* however, and the specialised pollination strategy of this species presents a challenge for using seed stock.

More than any other species, obligate hyperaccumulator plants that have a restricted distribution on isolated ultramafic outcrops are susceptible to habitat degradation (Galey et al. 2017). *Phyllanthus rufuschaneyi* is known only from a site that has been severely affected by over-logging and (man-made) forest fires for clearing of agricultural land, and neither the area nor the species has statutory protection. The current expansion of oil palm plantations on Lompoyou Hill cast a further shadow over its continued existence in the wild. As such, the case of *P. rufuschaneyi* highlights the importance of habitat and germplasm preservation if rare species are to be used in green technologies such as agromining. Therefore, concerted efforts must be made to screen for hyperaccumulator species (Whiting et al. 2004), followed by appropriate methods for conserving them both in and ex situ (Erskine et al. 2012). The utilization of *P. rufuschaneyi* in agromining operations means that there are now likely to be more plants in cultivation than in the wild, and this will for now safeguard its future survival.

## References

[CR1] Baker AJM, Proctor J, van Balgooy MMJ, Reeves RD (1992) Hyperaccumulation of nickel by the flora of the ultramafics of Palawan, Republic of the Philippines. In: Baker AJM, Proctor J, Reeves RD (ed) The vegetation of ultramafic (serpentine) soils. Proceedings of the first international conference on serpentine ecology. Intercept, Andover pp 291–304

[CR2] Bani A, Echevarria G, Zhang X, Benizri E, Laubie B, Morel J-L, Simonnot M-O (2015). The effect of plant density in nickel-phytomining field experiments with Alyssum murale in Albania. Aust J Bot.

[CR3] Beaman JH (2005). Mount Kinabalu: hotspot of plant diversity in Borneo. Biol Skr.

[CR4] Beaman JH, Beaman RS, Baas P, Kalkman K, Geesink R (1990). Diversity and distribution patterns in the flora of Mount Kinabalu. The plant diversity of Malesia.

[CR5] Beentje H (2016). The Kew plant glossary: an illustrated dictionary of plant terms.

[CR6] Chaney RL, Parr JF, Marsh PB, Kla JM (1983). Plant uptake of inorganic waste constituents. Land treatment of hazardous wastes.

[CR7] Chaney RL, Angle JS, Baker AJM, Li YM (1998) Method for phytomining of nickel, cobalt and other metals from soil. US Patent 5: 711–784, 27 January 1998

[CR8] Chaney RL, Baker AJM, Morel JL, Van der Ent A, Echevarria G, Baker AJM, Morel JL (2018). The long road to developing agromining/phytomining. Agromining: farming for metals. Mineral resource reviews.

[CR9] Echevarria G, Van der Ent A, Echevarria G, Baker AJM, Morel JL (2018). Genesis and behaviour of ultramafic soils and consequences for nickel biogeochemistry. Agromining: farming for metals. Mineral resource reviews.

[CR10] Erskine P, van der Ent A, Fletcher A (2012). Sustaining metal-loving plants in mining regions. Science.

[CR11] Galey MC, van der Ent A, Iqbal MCM, Rajakaruna N (2017). Ultramafic geoecology of south and southeast asia. Bot Stud.

[CR12] Govaerts R, Frodin DG, Radcliffe-Smith A (2000). World checklist and bibliography of Euphorbiaceae (with Pandaceae).

[CR13] Hoffmann P, Kathriararachchi H, Wurdack KJ (2006). A phylogenetic classification of Phyllanthaceae (Malpighiales; Euphorbiaceae sensu lato). Kew Bull.

[CR14] Hoffmann P, Baker AJM, Proctor J, Madulid D (2008). *Phyllanthus balgooyi* (Euphorbiaceae s.l.), a new nickel-hyperaccumulating species from Palawan and Sabah. Blumea.

[CR15] Homer F (1991) Chemical studies on some plants that hyperaccumulate nickel. Ph.D. thesis, Massey University, pp 1–285

[CR16] Huang L, Bell RW, Dell B, Woodward J (2004). Rapid nitric acid digestion of plant material with an open-vessel microwave system. Commun Soil Sci Plant Anal.

[CR17] IUCN (2001). IUCN red list categories and criteria: version 3.1.

[CR18] Jaffré T (1977) Composition chimique élémentaire des tissus foliaires des espèces végétales colonisatrices des anciennes mines de nickel en Nouvelle Calédonie. Cah. O.R.S.T.O.M., sér. Biol. XII: 323–330

[CR19] Jaffré T, Brooks RR, Lee J, Reeves RD (1976). *Sebertia acuminata*: a hyperaccumulator of nickel from New Caledonia. Science.

[CR55] Jaffré T, Pillon Y, Thomine S, Merlot S (2013). The metal hyperaccumulators from New Caledonia can broaden our understanding of nickel accumulation in plants. Front Plant Sci.

[CR20] Kathriarachchi H, Samuel R, Hoffmann P, Mlinarec J, Wurdack KJ, Ralimanana H, Stuessy TF, Chase MW (2006). Phylogenetics of tribe Phyllantheae (Phyllanthaceae; Euphorbiaceae *sensu lato*) based on nrITS and plastid matK DNA sequence data. Am J Bot.

[CR21] Kato M, Kawakita A (2017). Obligate pollination mutualism.

[CR22] Kawakita A (2010). Evolution of obligate pollination mutualism in the tribe Phyllantheae (Phyllanthaceae). Plant Species Biol.

[CR23] Kawakita A, Kato M (2004). Evolution of obligate pollination mutualism in New Caledonian *Phyllanthus* (Euphorbiaceae). Am J Bot.

[CR24] Kersten W, Brooks R, Reeves R, Jaffré T (1979). Nickel uptake by New Caledonian species of *Phyllanthus*. Taxon.

[CR25] Kersten WJ, Brooks RR, Reeves RD, Jaffré A (1980). Nature of nickel complexes in *Psychotria douarrei* and other nickel-accumulating plants. Phytochemistry.

[CR26] Leon B, Alain B (1953) Flora de Cuba. III. Euphorbiaceae. Contribuciones Ocasionales del Museo de Historia Natural del Colegio de la Salle 13:44–59

[CR27] Li Y-M, Chaney R, Brewer E, Roseberg R, Angle JS, Baker A, Reeves R, Nelkin J (2003). Development of a technology for commercial phytoextraction of nickel: economic and technical considerations. Plant Soil.

[CR29] Mesjasz-Przybylowicz J, Przybylowicz W, Barnabas A, van der Ent A (2016). Extreme nickel hyperaccumulation in the vascular tracts of the tree *Phyllanthus balgooyi* from Borneo. New Phytol.

[CR30] Montargès-Pelletier E, Chardot V, Echevarria G, Michot LJ, Bauer A, Morel J-L (2008). Identification of nickel chelators in three hyperaccumulating plants: an X-ray spectroscopic study. Phytochemistry.

[CR31] Nkrumah PN, Baker AJM, Chaney RL, Erskine PD, Echevarria G, Morel J-L, van der Ent A (2016). Current status and challenges in developing nickel phytomining: an agronomic perspective. Plant Soil.

[CR32] Nkrumah P, Echevarria G, Erskine, PD, van der Ent A, Sumail S (2017) First growth trial of tropical ‘metal shrubs’ to be used in economic agromining. In: Proceedings of the 9th international conference on Serpentine ecology, 5–9th June 2017. Tirana-Pogradec, Albania, p 169

[CR33] Nkrumah P, Echevarria G, Erskine PD, van der Ent A (2018). The discovery of nickel hyper-accumulation in *Antidesma montis*-*silam*: from herbarium identification to field re-discovery. Ecol Res.

[CR34] Pruesapan K, Telford IRH, Bruhl JJ, Van Welzen PC (2012). Phylogeny and proposed circumscription of Breynia, Sauropus and Synostemon (Phyllanthaceae), based on chloroplast and nuclear DNA sequences. Aust Syst Bot.

[CR35] Quimado MO, Fernando ES, Trinidad LC, Doronila A (2015). Nickel-hyperaccumulating species of *Phyllanthus* (Phyllanthaceae) from the Philippines. Aus J Bot.

[CR36] Reeves RD (2003). Tropical hyperaccumulators of metals and their potential for phytoextraction. Plant Soil.

[CR37] Reeves RD, Baker AJM, Borhidi A, Berazaín R (1996). Nickel-accumulating plants from the ancient serpentine soils of Cuba. New Phytol.

[CR38] Reeves RD, Baker AJM, Jaffré T, Erskine PD, Echevarria G, van der Ent A (2017). A global database for hyperaccumulator plants of metal and metalloid trace elements. New Phytol.

[CR39] Schmid M, Morat P, Mackee HS (1991). *Phyllanthus*. Flore de la Nouvelle-Calédonie et Dépendances.

[CR40] Van der Ent A, Mulligan D (2015). Multi-element concentrations in plant parts and fluids of Malaysian nickel hyperaccumulator plants and some economic and ecological considerations. J Chem Ecol.

[CR41] Van der Ent A, Baker AJM, Reeves RD, Pollard AJ, Schat H (2013). Hyperaccumulators of metal and metalloid trace elements: facts and fiction. Plant Soil.

[CR42] Van der Ent A, Baker AJM, van Balgooy MMJ, Tjoa A (2013). Ultramafic nickel laterites in Indonesia (Sulawesi, Halmahera): mining, nickel hyperaccumulators and opportunities for phytomining. J Geochem Explor.

[CR43] Van der Ent A, Mulligan D, Erskine PD (2013c) Discovery of nickel hyperaccumulators from Kinabalu Park, Sabah (Malaysia) for potential utilization in phytomining. In: Enviromine 2013, Santiago, Chile, 4–6 December 2013. pp 213–221

[CR44] Van der Ent A, Baker AJM, Reeves RD, Chaney RL, Anderson CWN, Meech JA, Erskine PD, Simonnot M-O, Vaughan J, Morel J-L (2015). Agromining: farming for metals in the future?. Environ Sci Technol.

[CR45] Van der Ent A, Erskine P, Sumail S (2015). Ecology of nickel hyperaccumulator plants from ultramafic soils in Sabah (Malaysia). Chemoecology.

[CR46] Van der Ent A, Repin R, Sugau J, Wong KM (2015). Plant diversity and ecology of ultramafic outcrops in Sabah (Malaysia). Aust J Bot.

[CR47] Van der Ent A, van Balgooy M, van Welzen P (2016). *Actephila alanbakeri* (Phyllanthaceae): a new nickel hyperaccumulating plant species from localised ultramafic outcrops in Sabah (Malaysia). Bot Stud.

[CR48] Van der Ent A, Echevarria G, Tibbett M (2016). Delimiting soil chemistry thresholds for nickel hyperaccumulator plants in Sabah (Malaysia). Chemoecology.

[CR49] Van der Ent A, Damien L, Callahan DL, Noller BN, Mesjasz-Przybylowicz J, Przybylowicz WJ, Barnabas A, Harris HH (2017). Nickel biopathways in tropical nickel hyperaccumulating trees from Sabah (Malaysia). Sci Rep.

[CR50] Van Welzen PC, Pruesapan K, Telford IRH, Esser H, Bruhl JJ (2014). Phylogenetic reconstruction prompts taxonomic changes in Sauropus, Synostemon and Breynia (Phyllanthaceae tribe Phyllantheae). Blumea.

[CR51] Vaughan J, Riggio J, Chen J, Peng H, Harris HH, van der Ent A (2017). Characterisation and hydrometallurgical processing of nickel from tropical agromined bio-ore. Hydrometallurgy.

[CR52] Webster GL (1956) A monographic study of the West Indian species of *Phyllanthus*. J Arnold Arbor 37: 91–122, 217–268, 340–359

[CR53] Whiting S, Reeves RD, Richards D, Johnson M, Cooke J, Malaisse F, Paton A, Smith J, Angle J, Chaney R (2004). Research priorities for conservation of metallophyte biodiversity and their potential for restoration and site remediation. Restor Ecol.

[CR54] Wu M-J, Huang T-C, Liu C-C, Chen Y-J, Chang Y-S, Hsu C-L, Wu S-Y, Tseng A-Y, Chang Y-C, Liu C-C, Kaewmuan A (2016). Pollen morphology and taxonomy in Malesian *Phyllanthus* (Phyllanthaceae). J Jpn Bot.

